# Cross-recurrence quantification analysis captures inter-brain coupling during naturalistic negotiation: a new dynamic approach for hyperscanning

**DOI:** 10.3389/fnins.2025.1713357

**Published:** 2026-01-12

**Authors:** Bear M. Goldstein, Shannon M. Burns, Fleming C. Peck, Rick Dale, Matthew D. Lieberman

**Affiliations:** 1 Department of Psychology, University of California, Los Angeles, CA, United States; 2 Department of Psychological Science and Neuroscience, Pomona College, Claremont, CA, United States; 3 Department of Communication, University of California, Los Angeles, CA, United States

**Keywords:** communication, CRQA, fNIRS, hyperscanning, negotiation, recurrence, social neuroscience, synchrony

## Abstract

Naturalistic interactions involve dynamic, nonlinear coordination that unfolds across multiple timescales, yet most hyperscanning studies rely on analytical methods designed for passive, stimulus-locked contexts. We introduce cross-recurrence quantification analysis (CRQA)—a method that treats signals as a coupled dynamical system and characterizes the patterns of their joint trajectories across time—to measure brain-to-brain coupling during a free-flowing negotiation task. Dyads were scanned with fNIRS as they negotiated budget allocations to solve a public health crisis. We measured neural coupling in regions critical for social cognition—medial prefrontal cortex and temporal parietal junction—and related coupling patterns to both objective negotiation behaviors and subjective experiences. While conventional measures of neural synchrony, such as inter-subject correlation and wavelet coherence, showed no relationships with outcomes, CRQA revealed systematic associations between dynamic coupling patterns and successful interaction. We found that balanced neural coordination, where leading and lagging flowed symmetrically between partners, predicted greater collaborative allocation adjustment and more positive social experiences, including shared understanding, cooperation, and liking. Longer delays in neurocognitive coordination, as opposed to immediate alignment, were associated with greater feelings of motivation during the interaction. Finally, greater complexity, or entropy, of neural coupling was linked to more parity in how partners moved toward the joint solution rather than a one-sided accommodation. These findings demonstrate that real social interaction can be captured through analytical methods that account for the dynamic, nonlinear processes being studied, creating new possibilities for understanding how minds connect during natural human interaction.

## 1 Introduction

Successful human collaboration is rarely a state of perfect synchronization. Instead, it is a dynamic process of mutual adjustment, where individuals continuously exchange information, integrate diverse perspectives, and converge on solutions. Whether negotiating a high-stakes contract or simply planning a dinner with friends, successful interaction relies on a reciprocal push and pull where partners lead, follow, and adapt to one another in real time. While this fluid coordination is fundamental to our social lives, its non-linear nature presents a significant challenge for neuroscientific study, as traditional analytical methods often struggle to capture the intricate, time-lagged dynamics that define natural interaction.

Recent advances in neuroimaging during social interaction—particularly hyperscanning approaches that simultaneously measure multiple brains (Montague et al., 2002; Dumas et al., 2011; Babiloni and Astolfi, 2014)—have made progress by demonstrating that we can capture neural processes during interaction (Redcay and Schilbach, 2019; Czeszumski et al., 2020; Hakim et al., 2023). This represents a major methodological advance, as hyperscanning offers continuous, unobstructed measurement of neural processes that support coordination in real time. These studies have examined how brains couple when people interact in, for example, cooperative games (Cui et al., 2012), joint problem solving (Fishburn et al., 2018), educational contexts (Dikker et al., 2017), and open discussion (Jiang et al., 2015). Synthesizing this work, recent reviews and meta-analyses have demonstrated that cooperative and communicative interactions are often accompanied by inter-brain synchrony, particularly in prefrontal and temporoparietal regions (Czeszumski et al., 2020, 2022; Kelsen et al., 2022).

However, despite this ability, existing hyperscanning analytical methods lack the flexibility to account for the inherently dynamic and fluid processes that unfold between people as they interact. This constraint limits our power to apply and measure fully naturalistic paradigms (Hasson and Frith, 2016; Burns et al., 2025). In this paper, we implement a flexible approach to studying real-time interaction at the level of the brain, aligning the dynamical processes being measured with dynamical measurement tools.

Most hyperscanning studies rely on stationary or time-locked measures of synchrony, such as inter-subject correlation (ISC; Hasson et al., 2004), phase synchrony (Lachaux et al., 1999), or wavelet coherence (Grinsted et al., 2004). While these approaches effectively capture coordinated responses to external stimuli, they were developed for passive, stimulus-driven designs (e.g., watching a movie). When applied to interactions involving reciprocal exchange and flexible turn-taking, these methods risk missing the nonlinear, time-lagged coordination that supports conversational dynamics (Hasson and Frith, 2016; Holroyd, 2022; Burns et al., 2025). For example, a negotiation between two people involves complex patterns of information flow that unfold at multiple timescales, where an influential argument might resonate seconds after being articulated as the listener integrates it with existing knowledge. This is the kind of delayed and asymmetric coupling that stationary synchrony measures can overlook (Marwan et al., 2007; Wallot and Leonardi, 2018).

**Figure 1:**
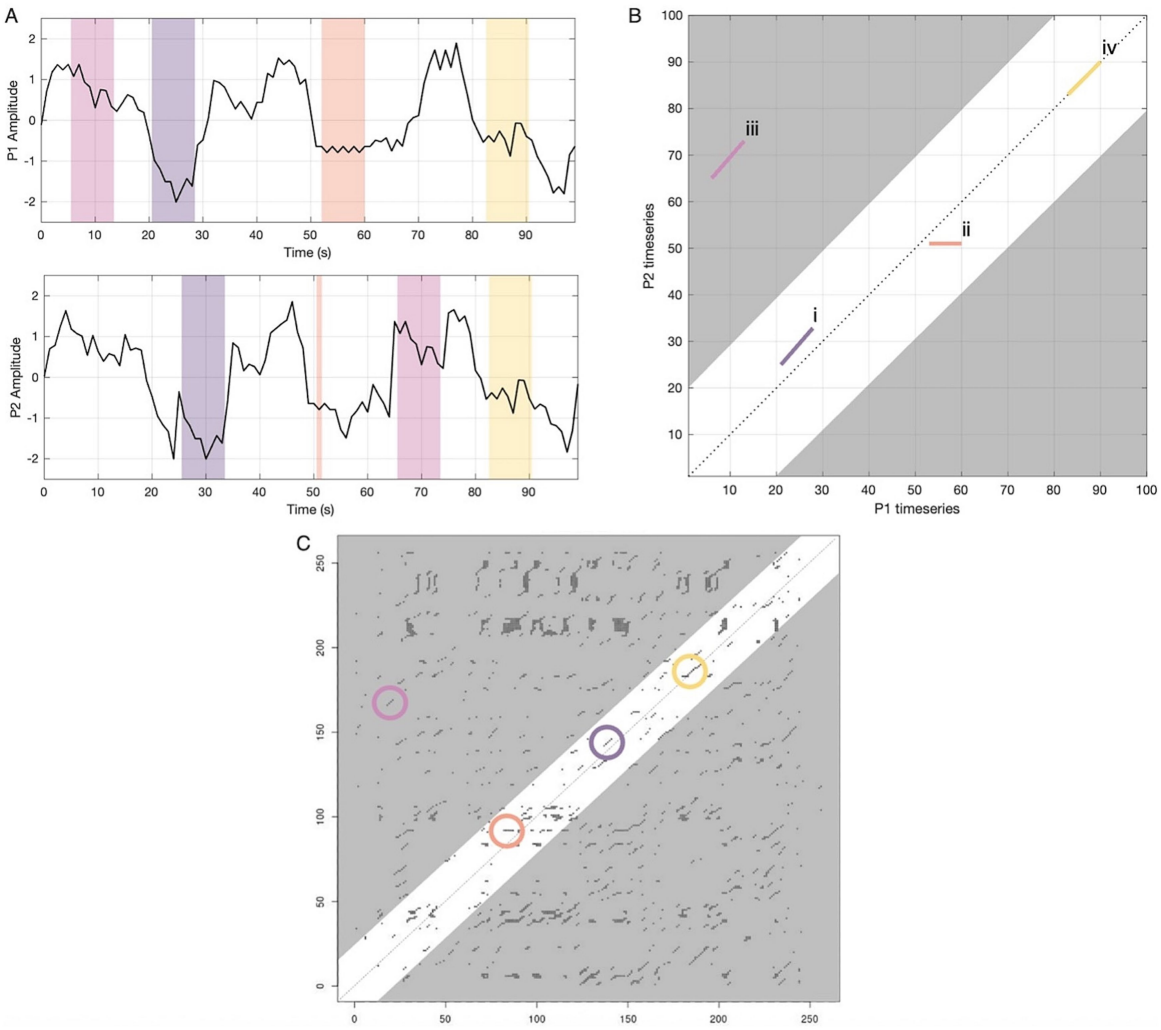
A model of CRQA for dynamic neural coupling during interaction. This figure illustrates how continuous neural signals are transformed into a visual representation of their temporal relationship. The panels on the top left **(A)** show hypothetical neural timeseries in a particular neural region from two conversation partners, with colored regions highlighting different types of recurrence, or coupling, across time. The top right panel **(B)** illustrates visual patterns of overlapping states in a hypothetical cross-recurrence plot (CRP) that would emerge from that data, with the *x*-axis corresponding to participant 1’s timeseries and the *y*-axis corresponding to participant 2’s. Each colored segment (i–iv) is the CRP counterpart of the time windows of the same color in the timeseries panels. For simplicity, only four lines are illustrated in this CRP. In a full analysis, CRQA measures all such patterns in time across all possible time lags and so full CRP plots tend to be more complex. Additionally, the 100-s period in these panels is for illustrative purposes only; the real data varied in length and the entire timeseries were analyzed. The bottom panel **(C)** shows a real CRP from the present study. Each point on the real plot **(C)** corresponds to a brief synchronized response between P1 and P2. Selective episodes of recurrence that correspond to the dynamics displayed in 1a-b are circled in their respective colors. Descriptions of dynamics in panels A/B: *(i) Diagonal deterministic structure (purple).* Here P2 inhabits the same sequence of neural activity as P1, with a delay or lag of about 5 s. On the CRP this appears as an off-diagonal line parallel to the main diagonal. The proportion of recurrent points forming these diagonal structures reflects the system’s predictability and structured co-evolution (Determinism). Variability in line lengths captures coordination complexity and chaos (Entropy). *(ii) Flat laminar structure (orange).* Here P1 becomes “trapped” in a relatively stable state that P2 was also in, but has since shifted out of. On the CRP this yields a horizontal line (or a vertical line if roles were reversed). The proportion of points forming these horizontal and vertical structures (Laminarity) and their average duration (Trapping Time) quantify the temporal stability of shared neural configurations. *(iii) Distant recurrence (pink) and band around the diagonal.* P2 later revisits a pattern P1 expressed much earlier. This produces a diagonal line far from the main diagonal, but such distant echoes may not be meaningful or interpretable—neurocognitive coordination likely occurs within shorter timeframes. By focusing analysis within a theoretically motivated band around the main diagonal (±20 s, white region), we exclude potentially spurious long-range correlations (gray regions). *(iv) No-lag synchrony (yellow).* P1 and P2 display an episode of in-the-moment synchronization as their neural activity follows the same trajectories simultaneously. This kind of alignment is analogous to what traditional linear methods (e.g., ISC) capture. CRQA captures these coupling dynamics as well, which result in recurrence lines directly on the main diagonal, also called the line of synchronization (LOS).

Cross-recurrence quantification analysis (CRQA) is well suited to address this methodological gap. CRQA is a nonlinear timeseries analysis that treats two interacting signals as a coupled dynamical system and characterizes the patterns of their joint trajectories (Shockley et al., 2002, 2009). Rather than measuring covariance or coherence, CRQA detects moments when one person’s brain activity is in a similar state to their partner’s activity at any point across an interaction, making it sensitive to delayed or irregular patterns that prior methods would miss. Because it can capture both covariance and other more irregular patterns across time, CRQA can be regarded as a nonlinear generalization of correlation and can pick up on sudden and abrupt shifts in behavior (Trulla et al., 1996; Marwan et al., 2007; Wallot et al., 2013), including loosely coupled systems such as two people interacting (Dale et al., 2011). CRQA measures are computed from a visualization referred to as a cross-recurrence plot that depicts how two systems (e.g., brains) revisit similar states over time without assuming stationarity or fixed temporal relationships (Marwan et al., 2007; Marwan, 2008). From these plots, CRQA derives a series of measures that reflect different aspects of coupling dynamics, including the predictability of coordination (‘Determinism’), the complexity of coordination patterns (‘Entropy’), and the temporal stability of shared neural states (‘Laminarity’ and ‘Trapping Time’). See Figure 1 for an illustration and further explanation of these dynamics.

**Figure 2:**
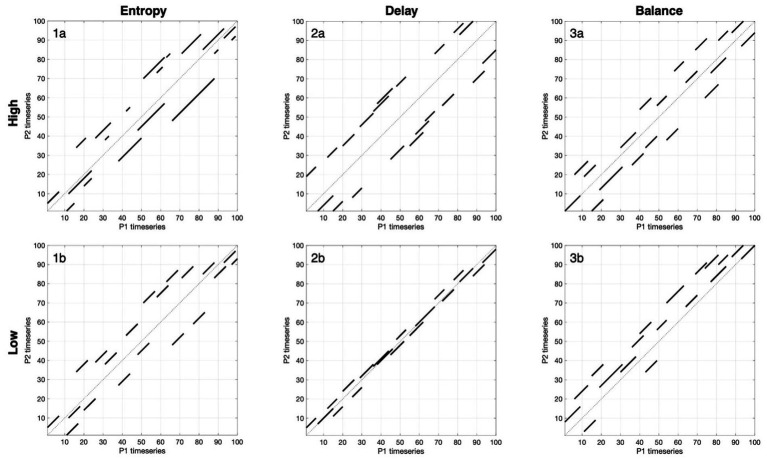
Visual guide to three CRQA metrics for neural coupling during conversation. This figure provides a visual guide to interpreting the three key metrics used in our results – entropy, delay, and balance – by modeling “high” and “low” examples. Each panel shows a simplified cross-recurrence plot for three focal measures. Only diagonal lines within the ±20-s band around the LOS are displayed. These examples demonstrate how CRQA characterizes not only whether brains couple but also how they couple through the complexity (entropy), timescale (delay), and reciprocity (balance) of neural coupling during interaction. **(1a,b)** Entropy reflects the variability of diagonal line lengths, capturing coordination complexity. High entropy (1a) shows diverse line lengths, reflecting flexible coordination patterns where partners may recur briefly, then for extended periods, then briefly again to varying degrees, creating a complex temporal structure. Low entropy (1b) shows uniform line lengths, indicating that when partners inhabit recurrent states, they do so for consistently similar durations. **(2a,b)** Delay measures the temporal offset at which recurrence occurs. High delay (2a) shifts lines consistently away from the diagonal, showing that partners coordinate reliably but with longer lags (for example, one partner following seconds later). Low delay (2b) has lines clustered tightly around the main diagonal, indicating near-simultaneous alignment. **(3a,b)** Balance captures symmetry in coordination. High balance (3a) distributes lines more evenly across both sides, suggesting influence alternates and neither partner consistently leads or lags. Low balance (3b) places most lines on one side of the diagonal, meaning one partner tends to lead neurally while the other lags.

CRQA also offers the flexibility to derive novel measures specifically geared toward studying conversation Wallot et al. (2013). For example, CRQA can be applied focally to a selective band around the main diagonal to capture near-real-time coupling, excluding distant temporal echoes less relevant to conversational coordination. Within this band, one can explore distinctive aspects of two-brain coupling, such as the typical time offset of neural alignment (‘Delay’), which reveals whether partners coordinate in real-time or with systematic lags, and the symmetry of coordination flow (‘Balance’), which indicates whether one person consistently leads or lags, or whether influence alternates dynamically. These measures move beyond asking whether signals are synchronized to reveal how they are dynamically coupled, making CRQA ideally suited to capture the temporal complexity of naturalistic conversation in contexts like negotiation. See Figure 2 for illustrations of select CRQA measures; see Supplementary material S1 for a table comparing CRQA to more conventional methods.

CRQA has proven valuable across multiple domains of interpersonal coordination. In behavioral research, CRQA has been used to quantify coupling in postural sway, gaze patterns, conversational turn-taking, and physiological synchrony during social interaction (Shockley et al., 2003; Richardson and Dale, 2005; Fusaroli and Tylén, 2016; Palumbo et al., 2017). Applications in neuroscience remain limited, primarily focusing on single-brain analyses to distinguish clinical states or cross-regional connectivity within individuals (Leonardi and Van De Ville, 2015; Pentari et al., 2023). Only one conference paper has extended CRQA to two brains, and even then in a constrained finger-tapping task with a confederate (Scheurich et al., 2019). To our knowledge, no experiments have leveraged the potential of CRQA to measure the flexible, reciprocal neural dynamics that unfold during social interaction.

In this study, we apply CRQA to measure neural coupling during naturalistic negotiation and decision-making. The purpose is twofold. Conceptually, we test whether patterns of neural coupling during conversation relate to both objective decision-making outcomes and subjective experiences of collaboration quality. Methodologically, we evaluate CRQA as a tool for capturing these dynamics in ways that complement or exceed conventional linear coupling metrics.

We recorded fNIRS data as dyads engaged in a free-flowing negotiation task, allocating a hypothetical $100 million budget across five organizations to address a public health crisis. This task exemplifies the complex, real-world decision-making that teams regularly face, requiring partners to exchange perspectives on priorities, discuss trade-offs between competing objectives, and ultimately converge on a shared allocation strategy through unstructured dialog. We leveraged the superior portability, motion tolerance, and comfort of fNIRS to study people as they interact naturalistically.

Given the social nature of our negotiation task, we focused our analysis on the medial prefrontal cortex (mPFC) and bilateral temporal parietal junction (TPJ). Specifically, the mPFC has been linked to representing the value of outcomes for self and others, integrating social and contextual information into choice, and supporting cooperative decision-making (Euston et al., 2012; Schuck et al., 2016; Zoh et al., 2022). Relatedly, the TPJ has been consistently implicated in theory of mind, joint attention, and flexible shifts in perspective, as well as in how people construe information (Saxe and Kanwisher, 2003; Schurz et al., 2014; Krall et al., 2015; Lieberman, 2022). Together, these default mode network hubs have shown to be central to social cognition and subjective construal processes, supporting the mentalizing, perspective-taking, and conceptual integration essential for successful negotiation and joint decision-making (Lieberman et al., 2019; Czeszumski et al., 2022; Kelsen et al., 2022).

While this study represents the first application of CRQA to naturalistic brain-to-brain coupling, we formed initial expectations based on the demands of negotiation and decision-making. Broadly, we expected that CRQA’s sensitivity to dynamic, time-varying coordination would reveal systematic relationships between neural coupling and both objective behavioral indicators (e.g., how much people changed their original stances, and how one-sided the change was) as well as subjective experiences of the interaction (e.g., perceived cooperation, partner quality, interpersonal liking, motivation, shared understanding, and satisfaction). We formed three specific predictions:

*H*1: We hypothesized that better collaborative outcomes and experiences would be characterized by more flexible and varied coordination patterns (‘Entropy’) as partners converge on a shared decision.

*H*2: We hypothesized a positive relationship between outcomes and longer coordination lags (‘Delay’), reflecting the effort of integrating differing perspectives rather than settling for a rushed, superficial agreement.

*H*3: Finally, we hypothesized that more evenly distributed neural coordination (‘Balance’)—where neither partner consistently lags or leads the other—would be associated with better outcomes.

Together, the dynamical measures made possible by CRQA should illuminate relationships between collaborative decision-making and brain-to-brain coupling that conventional synchrony methods may miss.

## 2 Materials and methods

### 2.1 Participants

Participants (*N* = 229, mean age = 20.32 years, SD = 2.60) were recruited from a large California university student population. After attrition, 220 participants were paired into 110 same-gender dyads (71 female–female, 39 male–male). The sample was ethnically diverse: 63 White/Caucasian (28.64%), 49 Biracial/Mixed (22.27%), 48 Hispanic/Latinx (21.82%), 42 Asian/Pacific Islander (19.09%), 10 Middle Eastern/North African (4.55%), 7 Black/African-American (3.18%), and 1 Native American (0.45%). Following data collection, dyads were excluded for technical issues with fNIRS data collection (*n* = 4) and failing to reach a 3-min conversation minimum (*n* = 5), yielding 101 dyads with viable neural and subjective experience data. An additional 5 dyads were excluded from behavioral analyses only due to missing or unreadable task responses, resulting in 96 dyads with complete behavioral, neural, and subjective data. All procedures were approved by the institutional review board (IRB#16–001901), and participants provided informed consent.

### 2.2 Procedure

**Figure 3:**
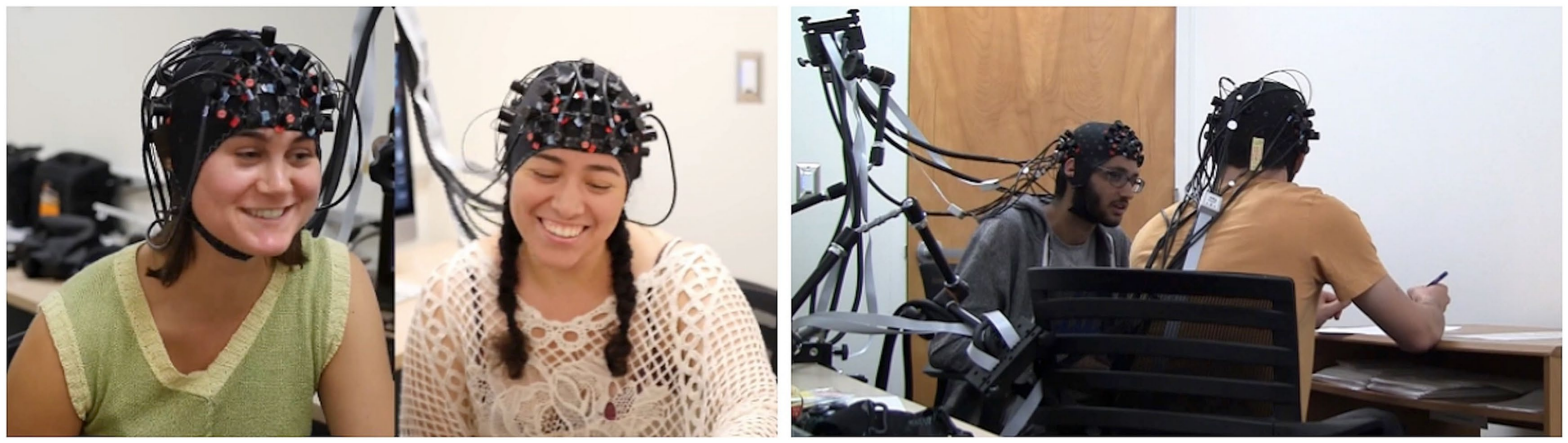
Stills from the experiment. Participants complete the joint resource-allocation task while wearing fNIRS caps.

The experimental procedure was modeled after the joint resource allocation task described in Keltner and Robinson (1993). Prior to laboratory sessions, participants completed a political identity questionnaire and were randomly paired with respect to their political positions. Upon arrival, participants were welcomed to separate rooms and completed an individual resource allocation task. They were then brought together, fitted with fNIRS equipment, and instructed to complete the same allocation task jointly, arriving at a mutually agreed-upon solution. Participants were asked to discuss the issue in depth as if making decisions about real money allocation. Experimenters activated recording equipment and left the room for the duration of the discussion. Conversations were unconstrained in length, after which they were separated to complete post-discussion questionnaires. The task was audio-video recorded (Figure 3).

### 2.3 Materials

#### 2.3.1 Resource allocation task

Participants allocated a hypothetical $100 million among five programs addressing the Zika virus epidemic: (1) Scientific research and development to find a vaccine, (2) Public education about how best to avoid contracting the disease, (3) Research and development for improving the life quality of babies already born with microcephaly, (4) Subsidize health care for affected families, and (5) Research and implement mosquito control/eradication strategies. Discussion durations ranged from 180 s to 1868 s, with a mean duration of 516.33 s (SD = 290.31).

#### 2.3.2 Behavioral measures

We calculated two behavioral measures that captured different aspects of their negotiation process based on each partner’s individual pre-discussion allocations and the dyad’s joint allocation. First, we measured the ‘total stance movement’ that occurred during discussion by calculating how far each person moved from their initial individual position to reach the joint decision. For each partner, we calculated the sum of absolute changes across all five allocation categories, then summed both partners’ changes to capture how much the dyad adjusted their stances overall. Second, we measured how evenly those changes were shared between partners (‘stance movement parity’). This measure is high when both partners moved a similar amount from their initial stances and low when one partner did most of the adjusting while the other remained relatively unchanged. These measures were computed across each dyad’s five allocation decisions and summed to yield single values per dyad.

#### 2.3.3 Post-negotiation questionnaires

After their discussion, participants rated their experience using Likert scales across various social and performance dimensions. We created composite measures by averaging related items and then combining both partners’ responses to create dyad-level scores. Six composites were created that showed strong internal reliability. Cooperation captured perceptions of partner collaboration and willingness to work together (Cronbach’s *α* = 0.819); Partner Quality reflected evaluations of the partner’s contributions and ideas (α = 0.794); Liking measured personal rapport, comfort, and interest in future collaboration (α = 0.826); Motivation assessed how engaged both partners were in finding good solutions (α = 0.793); Shared Understanding evaluated whether participants believed they had a common grasp of the problem and solution approach (α = 0.763); and Satisfaction measured contentment with the final decision and solution quality (α = 0.847). Complete questionnaire items and composites can be found in Supplementary material S2.

#### 2.3.4 Neural data acquisition

**Figure 4:**
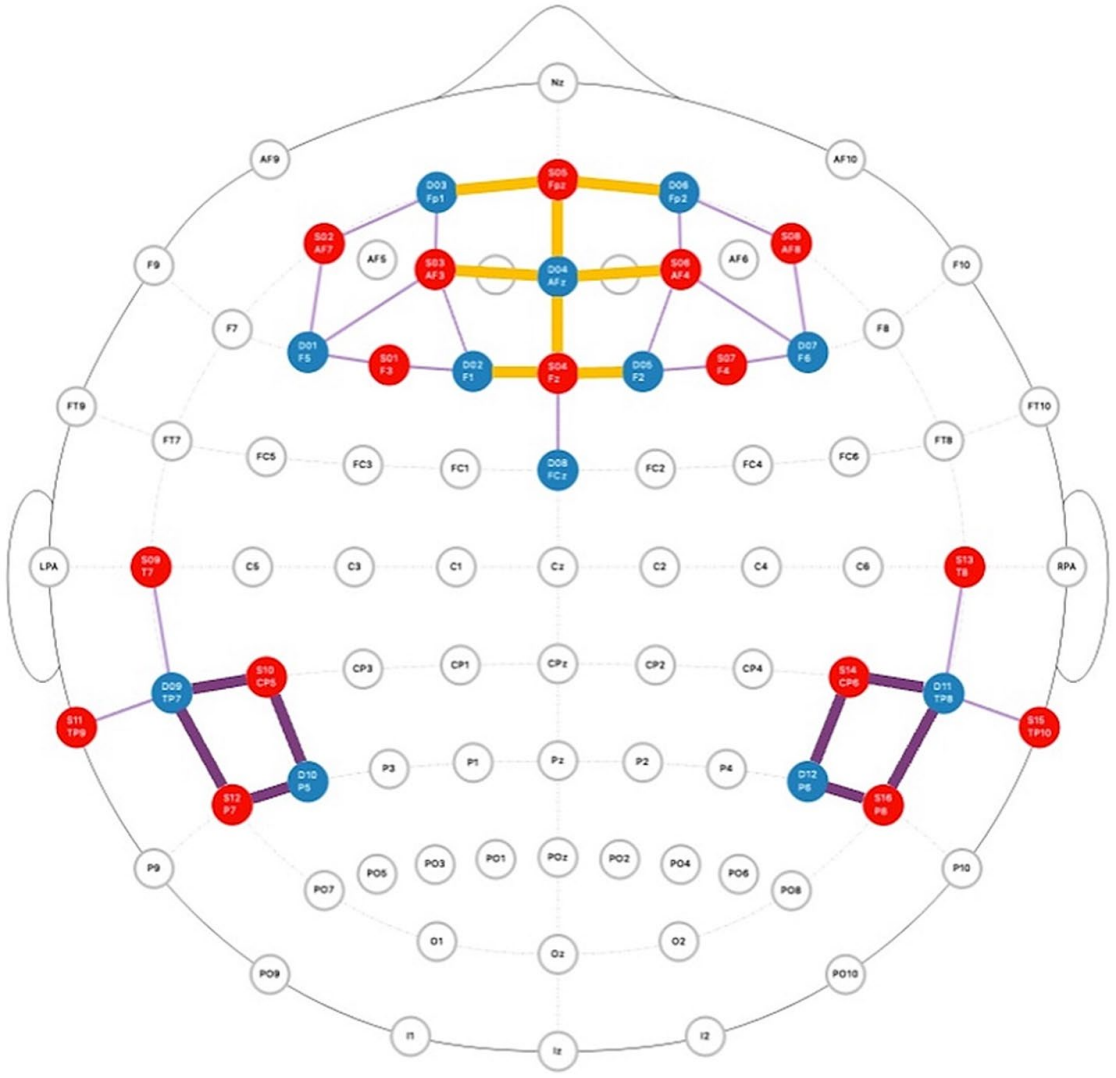
Montage of fNIRS probes across the prefrontal cortex and temporal parietal junction. The red dots represent source optodes and the blue dots represent detector optodes. ROIs are highlighted in orange (mPFC) and purple (bilateral TPJ).

Neural activity was recorded using functional near-infrared spectroscopy (fNIRS) with a NIRScout imaging unit (NIRx Technologies). The system employed 32 source and 32 detector optodes, split evenly between dyad members and secured in stretchy head caps. Optodes were positioned over the prefrontal cortex and bilateral temporal parietal junction using the 10–10 international positioning system, creating 35 measurement channels per participant (Figure 4). These positions corresponded to default mode network areas accessible in the cortex and commonly implicated in communication-related neural coherence. Light intensity data were collected at wavelengths of 760 nm and 850 nm with a sampling rate of 3.91 Hz.

### 2.4 Neural data preprocessing and CRQA

#### 2.4.1 Neural data preprocessing

fNIRS data underwent a standardized preprocessing pipeline (Yücel et al., 2021): (1) automatic identification and removal of noisy channels using frequency spectral analysis, (2) regression of motion and speaking confounds, (3) detection of motion artifacts (>5 standard deviation changes in <1 s), (4) rescaling of artifact segments to remove non-neural discontinuities, (5) bandpass filtering (0.008–0.2 Hz), (6) conversion of light intensity to relative change in hemoglobin concentration using the Modified Beer–Lambert Law, and (7) final evaluation and rejection of channels with remaining artifacts. Motion and speaking regression was performed early in the pipeline to remove patterns attributable to physical speech acts rather than underlying neural representations. For each dyad, we then extracted the conversation segment of oxygenated hemoglobin (HbO) fNIRS signals, z-scored per channel, and resampled to 1 Hz to smooth the signal. Within each anatomical region of interest (ROI), we averaged the relevant channels at each timepoint to form an ROI timeseries per person.

#### 2.4.2 CRQA

We applied cross-recurrence quantification analysis using the R package *crqa* (Coco and Dale, 2014; Coco et al., 2021, 2025) to quantify temporal coordination patterns between conversation partners. This approach reconstructs the dynamics of each partner’s neural activity into a phase space using a time-delay embedding, where each point represents the evolving state of the system, and identifies moments when both partners visit similar states. A recurrence point is defined as a pair of moments where partners’ neural patterns are close enough together in state space, represented by the formula:


$$R_{\mathrm{ij}}=\{\begin{array}{c} 1,\text{if}\;∥x_{i}-y_{j}∥₂\leq \varepsilon ,\\ 0,\text{if}\;∥x_{i}-y_{j}∥₂>\varepsilon \end{array}$$

For each pair of time points (i, j), the Euclidean distance between the partners’ embedded neural state vectors x_i_ and y_j_ is computed. If this distance is less than or equal to a dyad-specific radius ε, R_ij_ = 1 (a recurrence point), otherwise R_ij_ = 0.

We used standard parameters: Euclidean distance as our similarity metric, an embedding dimension of 2, and minimum line length of 2 for both diagonal and vertical structures. The analysis yielded standard CRQA outputs (e.g., determinism, entropy, laminarity, trapping time) as defined in the package documentation.

Following standard procedure to ensure consistent comparisons across dyads, we calibrated the recurrence threshold (radius) separately for each dyad and brain region to achieve approximately 3.5% recurrence rate—within the optimal 2–5% range recommended for continuous timeseries (Coco et al., 2025). We recorded each radius value and applied it consistently across all subsequent analyses for that dyad-region combination.

Because psychological coordination in dialog does not occur over an unlimited window, we focused our analysis on recurrence near the main diagonal, which represents real-time synchronization. After computing the full recurrence plot at the calibrated radius, we retained only recurrent points within ±20 s of the diagonal and set all others to zero. We then recalculated CRQA measures on this masked plot. This approach yielded band-limited metrics that specifically capture relevant coupling while excluding distant temporal echoes that are less relevant to conversational coordination.

##### 2.4.2.1 Diagonal cross-recurrence profile and lag-sensitive summaries

To understand the temporal dynamics of coordination—particularly who tends to lead and the typical time offset of alignment—we computed a diagonal cross-recurrence profile within the same ±20-s band also using the *crqa* package in R. For each possible diagonal lag off the main diagonal, this profile shows how often one partner’s brain state lined up with the other’s brain. We counted recurrent points along each diagonal lag and converted these to recurrence rates. We designed novel measures to characterize these profiles. ‘Delay’ represents how far apart in time partners tend to be when they align. It is calculated by taking the average of the absolute lags at which recurrence occurs, weighted by how much recurrence we observe at each lag. ‘Balance’ quantifies the symmetry of the coordination—whether coordination was one-sided or shared. It is calculated by totaling all recurrence on one side of the diagonal and all the recurrence on the other side, then taking the absolute difference divided by their sum and subtracting that value from 1. Higher values indicate greater balance on a 0–1 scale.

#### 2.4.3 Primary CRQA outcomes for analysis

While the full suite of CRQA measures mentioned above is available, our theoretical focus on flexible, short-lag alignment during naturalistic conversation led us to emphasize three key metrics computed within the ±20-s band: normalized Entropy, or rENTR, which captures the complexity and diversity of coordination patterns while controlling for the total number of recurrent structures; Delay, which indicates how close or distant in time alignment is concentrated; and Balance, which quantifies the symmetry of their coordination.

### 2.5 Analysis

#### 2.5.1 Primary analyses

We examined whether neural coordination patterns during conversation related to both behavioral task outcomes and subjective experiences. All analyses were conducted at the dyad level, focusing on our two *a priori* ROIs, mPFC and TPJ. For each brain region, we computed partial Spearman correlations between each neural coupling metric and predictors from two pre-specified families: behavioral measures (total stance movement and stance movement parity) and post-interaction questionnaire composites (cooperation, partner quality, liking, motivation, shared understanding, satisfaction). We used Spearman correlations to accommodate non-normal distributions and meaningful outliers and to capture monotonic relationships common in naturalistic dyadic data.

Conversations were unconstrained in length to allow for natural negotiation dynamics. The entire conversation was analyzed for each dyad, and we controlled for conversation duration in all analyses. Partialling out conversation duration ensured that associations reflected neural coordination dynamics rather than potential confounding effects of conversation length.

#### 2.5.2 Statistical significance and multiple comparisons control

To control family-wise error while accounting for dependencies among related tests, we implemented a Westfall-Young step-down max-statistic procedure (Westfall and Young, 1993; Nichols and Holmes, 2001) within each neural-metric and predictor-family combination. This approach involved generating a single set of permutations shared across all tests within each block, computing the maximum absolute statistic across the block for each permutation, and deriving adjusted *p*-values by comparing our observed statistics to the 1,000-permutation distribution using a step-down procedure. The behavioral outcome block consisted of 4 tests (2 behavioral measures x 2 ROIs); the subjective experience block consisted of 12 tests (6 composite self-report measures x 2 ROIs). We assessed significance at adjusted *α* = 0.05. This framework provides family-wise error control under minimal assumptions and accommodates correlated tests.

#### 2.5.3 ISC and WTC measures

As diagnostic comparisons, we computed two standard neural coupling metrics from the same conversation segments and ROI-averaged HbO timeseries used for CRQA (1 Hz, z-scored, and averaged across channels within each ROI for each person). For each dyad and ROI (mPFC, TPJ), we derived (a) an inter-subject correlation (ISC; Hasson et al., 2004) value that captures zero-lag linear synchrony and (b) a wavelet transform coherence (WTC; Grinsted et al., 2004) value that captures time-frequency-localized coupling. These measures served as familiar benchmarks to contextualize our primary CRQA-based findings rather than as primary outcomes.

To calculate ISC, we treated each dyad’s two ROI timeseries as paired observations over time. We first identified timepoints where both partners had finite values and computed the Pearson correlation coefficient between the two vectors. This yielded a single ISC value per dyad and ROI, reflecting the degree of zero-lag covariation between partners’ neural activity.

To calculate WTC, we used MATLAB’s *wcoherence* function to compute a time-frequency map of magnitude-squared coherence between partners’ ROI timeseries, again after excluding any timepoints where either signal was missing. We set the sampling rate to 1 Hz and used the default complex wavelet implemented in *wcoherence.* Specifically, we extracted the time-frequency coherence map, restricted analysis to the 0.02–0.08 Hz band, masked values outside the cone of influence to avoid edge artifacts, and computed the mean coherence across time and frequency. This measure summarizes the overall degree of low-frequency, time-localized coupling between partners’ neural signals during the conversation.

## 3 Results

**Figure 5:**
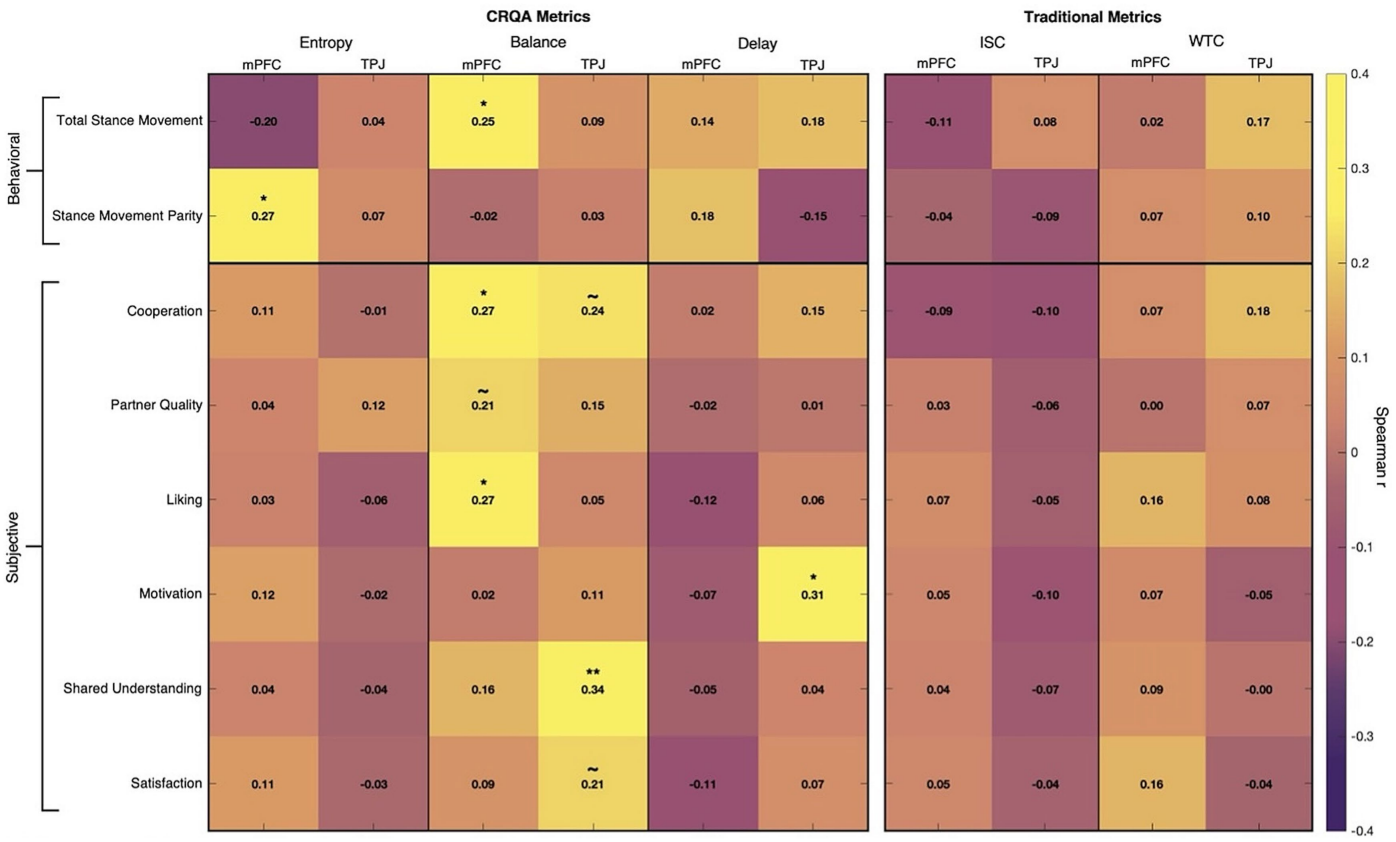
Results heatmap. Dyad-level partial Spearman correlations (controlling for conversation length) between collaboration outcomes (rows) and neural coupling metrics (columns). Left panel shows CRQA measures (entropy, balance, delay) for mPFC and TPJ; right panel shows traditional measures (ISC, WTC) for the same regions. Behavioral measures (total stance movement, stance movement parity) appear in the top section; subjective experience composites (cooperation, partner quality, liking, motivation, shared understanding, satisfaction) appear below. Color intensity reflects correlation strength. Overall, CRQA measures show multiple significant associations with both objective and subjective outcomes, while traditional measures show minimal reliable associations. Significance markers reflect Westfall-Young step-down correction within measure families: *p < 0.05, **p < 0.01, and ~ indicates unadjusted *p* < 0.05 that did not survive correction.

Our analysis revealed several relationships between neural coordination dynamics and both behavioral outcomes and subjective experiences during negotiation and decision-making (Figure 5). All correlations reported are partial Spearman correlations, controlling for conversation duration to provide a clean test of neural coordination effects independent of scan length. We assessed significance at a max-statistic-adjusted threshold of *α* = 0.05 and report these adjusted values below unless otherwise specified.

### 3.1 Baseline coupling measures did not capture outcomes

ISC produced no significant correlations with any behavioral or subjective measures, suggesting that simple linear synchrony was not able to capture brain dynamics relevant to conversation outcomes. WTC showed only marginal associations with cooperation (*r* = 0.18, unadjusted *p* = 0.079, adjusted *p* = 0.523) and total stance movement (*r* = 0.17, unadjusted *p* = 0.092, adjusted *p* = 0.324), neither of which survived multiple comparisons correction.

### 3.2 Entropy linked to more parity in negotiation behavior

Entropy, measuring complexity of coordination between brains, in the mPFC was positively associated with stance movement parity, i.e., more equal allocation change between partners (*r* = 0.27, *p* = 0.043). This suggests that more varied neural coordination patterns may reflect more effective integration of partner perspectives, where partners evenly contribute to moving toward the final solution rather than having one person do all of the adjusting.

### 3.3 Delay associated with more motivated interaction

In the TPJ, longer Delay, indicating greater average coupling lags, was associated with higher self-reported motivation (*r* = 0.31, *p* = 0.014). Rather than indicating inefficiency, these longer temporal offsets may reflect more engaged deliberation where partners invest greater effort in processing and responding to each other’s ideas, leading to increased feelings of motivation to find good solutions. Alternatively, motivated partners might be more intentional about articulating complex ideas that take time to resonate, actively retrieving and integrating information from earlier in the conversation.

### 3.4 Balance reflected by more positive interpersonal and collaborative dynamics

More neural coordination Balance—where neither partner consistently leads the interaction but rather neural leadership and lag occur evenly between both partners—emerged as the strongest predictor of outcomes. In the mPFC, balance was linked to greater total stance movement during negotiation (*r* = 0.25, *p* = 0.046), higher ratings of cooperation (*r* = 0.27, *p* = 0.046), and greater interpersonal liking (*r* = 0.27, *p* = 0.048). In the TPJ, balanced coordination predicted more perceived shared understanding (*r* = 0.34, *p* = 0.006). These findings suggest that when neural influence flows symmetrically between partners, dyads not only engage in more thorough and cooperative deliberation but also develop better mutual understanding and rapport.

### 3.5 Preliminary evidence for coordination dynamics and social experiences

Several additional associations emerged at marginal significance levels (unadjusted *p* < 0.05; multiple comparison-adjusted *p* > 0.05). Determinism in the TPJ showed a negative association with satisfaction (*r* = −0.21, unadjusted *p* = 0.038, adjusted *p* = 0.296), suggesting that overly rigid coordination patterns may hinder positive experiences. Trapping time in the mPFC was positively associated with perceived partner quality (*r* = 0.22, unadjusted *p* = 0.026, adjusted *p* = 0.219), potentially indicating that sustained shared neural states foster positive partner evaluations. Balance showed additional positive associations with satisfaction (*r* = 0.21, unadjusted *p* = 0.032, adjusted *p* = 0.187), cooperation (*r* = 0.23, unadjusted *p* = 0.018, adjusted *p* = 0.117), and partner quality (*r* = 0.21, unadjusted *p* = 0.031, adjusted *p* = 0.187), supporting the primary Balance findings. These results are reported for completeness and did not survive correction for multiple comparisons.

## 4 Discussion

This study demonstrates that CRQA is a powerful tool for capturing dynamic aspects of neural coupling that unfold during naturalistic negotiation. CRQA was applied to fNIRS data recorded from dyads as they allocated a hypothetical budget across public health programs in a free-flowing conversation. While traditional measures of neural coupling—ISC and WTC—provided less insight into negotiation outcomes and experiences, CRQA revealed systematic relationships between neural coordination patterns and both objective behaviors and subjective collaboration quality. These findings suggest that the temporal complexity of brain-to-brain coupling during real conversation can benefit from analytical methods sophisticated enough to flexibly capture non-linear, time-lagged dynamics rather than just moment-to-moment covariation.

The specific patterns revealed by CRQA demonstrate consistent relationships between neural coupling and effective negotiation and decision-making. Balanced coordination, where neural influence occurred symmetrically between partners rather than being dominated by one person, in the TPJ predicted greater shared understanding, while in the mPFC it was associated with higher cooperation, interpersonal liking, and movement away from each partners’ initial positions to create a new joint solution. Additionally, the amount of coordination delay and lag in the TPJ was associated with higher motivation. Finally, more complex and varied coupling patterns (i.e., greater Entropy) in the mPFC was linked to more equitable sharing of positional changes between partners. Taken together, these findings suggest that effective neural coupling during collaboration requires reciprocal rather than one-sided neurocognitive influence, extended lag time for processing and integrating divergent perspectives rather than immediate synchronization, and flexible coordination patterns rather than rigid predictability.

Converging behavioral evidence supports this neural profile. Consistent with the notion of Balance, work on collective performance shows that shared influence benefits groups. For example, more equal turn-taking has been linked to higher collective intelligence (Woolley et al., 2010), and meta-analyses have found associations between shared leadership and stronger team effectiveness and confidence (Nicolaides et al., 2014; Wang et al., 2014). Furthermore, the value of delayed coupling aligns with the Motivated Information Processing in Groups model, which argues that high epistemic motivation leads partners to elaborate information and therefore possibly slow down for higher-quality decision (De Dreu et al., 2008). The reciprocal, iterative processes in which partners progressively align and co-construct meaning (Pickering and Garrod, 2004; De Jaegher and Di Paolo, 2007) reinforces the importance of measuring delayed neural coupling, as interlocutors push, retrieve, and reintegrate information to develop shared understanding. Finally, evidence that flexible, non-rigid coordination supports collaboration includes findings that linguistic entropy predicts better joint dialog performance (Fusaroli et al., 2012; Fusaroli and Tylén, 2016) and that irregular, non-repetitive behavioral dynamics are associated with better collaborative problem-solving (Amon et al., 2019; Eloy et al., 2019).

The significant associations between CRQA measures of neural activity and both behavioral and subjective outcomes support their utility for advancing understanding of brain-behavior relationships. For example, the present findings align with and extend our understanding of the established roles of the mPFC and TPJ in social cognition. The TPJ has been implicated in theory of mind, joint attention, and subjective construal (Saxe and Kanwisher, 2003; Schurz et al., 2014; Krall et al., 2015; Lieberman, 2025), positioning it to support the shared understanding and motivation processes we observed. Relatedly, the mPFC has demonstrated a role in valuation and decision-making, compromise strategies, and cooperation (Euston et al., 2012; Schuck et al., 2016; Zoh et al., 2022), aligning with its links to effective and equitable negotiation processes as well as positive interpersonal ratings in this study.

Hyperscanning research has previously demonstrated coupling in these regions during social interaction (Czeszumski et al., 2022), but the traditional measures of ISC and WTC capture only stationary relationships and may miss the temporal dynamics of more complex coordination. Unsurprisingly, then, our results showed that these measures failed to demonstrate significant correlations with behavioral and social outcomes, whereas CRQA measures captured multiple associations. This enhanced sensitivity means CRQA may be able to provide new insights into brain-behavior relationships, such as functional distinctions within the default mode network. For example, our results show region-specific coordination profiles consistent with the CEEing model (Lieberman, 2022): coupling in the TPJ predicted primarily subjective outcomes (shared understanding, motivation, satisfaction), suggesting its coordination reflects the integration of perspectives into shared construals, while mPFC coordination predicted both behavioral outcomes and social evaluations, consistent with its broader role in more effortful, reflective social processes (see also, Goldstein et al., 2025a).

This study serves as a first application of CRQA to naturalistic neural coupling, opening several important avenues for future research. First, the neural signatures of effective collaboration—Balance, Delay, and Entropy—should be tested across different interaction contexts, such as competitive bargaining, creative brainstorming, and hierarchical leader-follower dynamics. Second, systematic parameter exploration should attempt to optimize CRQA for neural hyperscanning applications. We chose conventional settings to facilitate comparability, but subsequent work should capitalize on the flexibility of CRQA parameters by varying the delay and embedding dimension (to capture higher-order temporal structure), minimum line length (to distinguish transient vs. sustained coupling), and the temporal band around the diagonal (±10–30 s to target different conversational timescales). Other important methodological advances include time-resolved or windowed CRQA (Galati et al., 2026) that aligns to specific conversational events as well as investigations of whether neural metrics correspond to linguistic and behavioral measures (e.g., does neural coupling balance relate to speaking balance between interlocutors?). Finally, future work could also integrate CRQA metrics with classification frameworks like multi-timepoint pattern analysis (MTPA; Goldstein et al., 2025b), which successfully handles the high dimensionality of naturalistic fNIRS data to better measure and predict psychological states.

Overall, the present study demonstrates that CRQA offers a broadly useful way to quantify neural coordination in dyadic paradigms, with advantages extending well beyond negotiation alone. By capturing reciprocal, time-lagged, and nonlinear coupling rather than stationary alignment, CRQA is well suited to naturalistic settings with free-form conversation and flexible turn taking—not just rigid, tightly scripted tasks. As the field moves toward more ecologically valid designs, CRQA can serve as a standard analytic tool across topics in social neuroscience, from negotiation and decision-making to friendship formation, education, or teamwork.

## Data Availability

The datasets presented in this study can be found in online repositories. The names of the repository/repositories and accession number(s) can be found at: Open Science Framework at: https://osf.io/fctzx/.
