# Supplementary material for: Cross-recurrence quantification analysis captures inter-brain coupling during naturalistic negotiation: a new dynamic approach for hyperscanning
Source: Front Neurosci. 2026 Jan 12;19:1713357. doi: 10.3389/fnins.2025.1713357 (PMC12833299; doi:10.3389/fnins.2025.1713357)
Supplement: Supplementary file 1 [file Data_Sheet_1.pdf]

Supplementary Material

1 Comparison of analysis methods

	**Inter-Subject Correlation (ISC)**	**Wavelet Transform Coherence (WTC)**	**Cross-Recurrence Quantification Analysis (CRQA)**
**Primary Calculation**	Pearson correlation between two time-series.	Cross-spectrum normalized by the power spectrum of two signals using wavelet decomposition.	Phase-space reconstruction of two signals to identify shared states (recurrence points) across all possible time points.
**Mathematical Basis**	Linear dependence (Covariance).	Linear dependence (Coherence) in time-frequency space.	Non-linear dynamical systems theory (phase space recurrence).
**Temporal Relationship**	Assumes simultaneous or fixed-lag synchronization across the entire time-series.	Captures time-localized synchrony in specific frequency bands.	Captures coupling at any time lag, allowing for variable delays, non-stationarity, and evolving temporal offsets.
**Directionality & Symmetry**	Does not distinguish directional dynamics: $r_{xy} = r_{yx}$	Magnitude-squared coherence is non-directional. Phase differences can be estimated to infer lead–lag relations, but interpreting these in strongly non-stationary signals can be challenging.	Metrics like ‘Balance’ or diagonal cross recurrence profiles can quantify leader-follower dynamics and asymmetries.

**Key Output Metrics**	Correlation coefficient (r).	Average Coherence (0 to 1); Phase relationships.	Multidimensional**.** For example, Entropy (complexity), Balance (symmetry), Delay (lag), Determinism (predictability), Laminarity (stability), Trapping time (duration of stasis).
**Ideal Use Case**	Participants viewing the same movie or listening to the same narrative (simultaneous alignment).	Joint action tasks (e.g., finger tapping, walking) or oscillatory processes where frequency-specific coupling is expected.	Unstructured dialogue, negotiation, or problem-solving involving turn-taking, flexible lags, and emergent coordination.
**Psychological Construct**	"Are we processing this input similarly?"	"Are we moving/oscillating in time together?"	"How are we interacting? Is the system stable? Who is leading? How complex is our coordination?"

2 Questionnaire information

2.1 Composites

All composites were computed as the mean of their items. Composites were computed when all items were present. If a composite included one of the items from the 5-point scale in the questionnaire, it was linearly rescaled to align with the 7-point scales. Finally, all composites were required to meet the conventional Cronbach's alpha threshold of 0.70.

Cooperation ($k=2$, Cronbach's $\alpha=0.819$): "My partner was very cooperative", "My partner seemed very willing to work together to decide on the solution"

Partner Quality ($k=2$, Cronbach's $\alpha=0.794$): "My partner had good ideas", "My partner's input was very useful"

Liking (k=3, Cronbach's α=0.826): "I liked my partner as a person", "I found the interaction to be comfortable", "I would want to work with my partner again in the future"

Motivation (k=2, Cronbach's α=0.793): "I was motivated to find the best solution to the Zika epidemic", "My partner was motivated to find the best solution"

Shared Understanding (k=5, Cronbach's α=0.763): "My partner and I had a common understanding of what the problem was", "My partner and I had a common understanding of how to solve the problem", "We solved the problem in a way we both agree on", "We drew conclusions together", "The reasons my partner gave for their opinions made sense to me"

Satisfaction (k=4, Cronbach's α=0.847): "I think our solution is the best solution to the problem at hand", "My partner thinks our solution is the best solution to the problem at hand", "I am satisfied with the decision we came up with", "My partner is satisfied with the decision we came up with"

2.2 Full Questionnaire

Participants completed the post-interaction survey immediately after the negotiation task. All items are displayed in their original format and order below.

	Strongly Disagree	Moderately Disagree	Slightly Disagree	Neither agree nor disagree	Slightly Agree	Moderately Agree	Strongly Agree
My partner was very cooperative	○	○	○	○	○	○	○
My partner's input was very useful	○	○	○	○	○	○	○
I liked my partner as a person	○	○	○	○	○	○	○
My partner seemed very willing to work together to decide on the solution	○	○	○	○	○	○	○
My partner had good ideas	○	○	○	○	○	○	○
The reasons my partner gave for their opinions made sense to me	○	○	○	○	○	○	○
I think my partner would agree with me on other important issues	○	○	○	○	○	○	○

I would want to work with my partner again in the future	○	○	○	○	○	○	○
I found the interaction to be comfortable	○	○	○	○	○	○	○
I thought the interaction was very difficult to get through	○	○	○	○	○	○	○
This interaction made me feel very stressed	○	○	○	○	○	○	○

Indicate how true or false you think each statement is. Please be honest with your answers.

	Very False	Somewhat False	Unsure	Somewhat True	Very True
I was motivated to find the best solution to the Zika epidemic	○	○	○	○	○
My partner was motivated to find the best solution	○	○	○	○	○
I listened carefully to my partner	○	○	○	○	○
My partner listened carefully to me	○	○	○	○	○
The inputs from my partner and I complimented each other	○	○	○	○	○
We drew conclusions together	○	○	○	○	○
My partner and I handled differences of opinions by addressing them directly	○	○	○	○	○

Indicate how much you agree with each statement below. Please be honest with your answers.

	Strongly Disagree	Moderately Disagree	Slightly Disagree	A little	Slightly Agree	Moderately Agree	Strongly Agree
My partner gave me equal say in the decision	○	○	○	○	○	○	○
I tried to give my partner equal say in the decision	○	○	○	○	○	○	○
My partner and I had a common understanding of what the problem was	○	○	○	○	○	○	○
My partner and I had a common understanding of how to solve the problem	○	○	○	○	○	○	○
My partner and I had very different background information about the topic at the start of the negotiation	○	○	○	○	○	○	○
My partner and I are very different types of people	○	○	○	○	○	○	○

Indicate how much you agree with each statement below. Please be honest with your answers.

	Strongly disagree	Moderately disagree	Slightly disagree	Neither agree nor disagree	Slightly agree	Moderately agree	Strongly agree
We solved the problem in a way we both agree on	○	○	○	○	○	○	○
I am satisfied with the decision we came up with	○	○	○	○	○	○	○
My partner is satisfied with the decision we came up with	○	○	○	○	○	○	○
I think our solution is the best solution to the problem at hand	○	○	○	○	○	○	○
My partner thinks our solution is the best solution to the problem at hand	○	○	○	○	○	○	○

What things were important to consider when solving the Zika issue? (please use complete sentences)

____

____

____

____

____

In what ways do you think your solution addressed these things? (please use complete sentences)

____

____

____

____

____

Have you met your partner before this study?

- ◯ Yes
- ◯ No

What do you think were your partner's political views, in general?

- ◯ Strongly Conservative
- ◯ Moderately Conservative
- ◯ Slightly Conservative
- ◯ Centrist
- ◯ Slightly Liberal
- ◯ Moderately Liberal
- ◯ Strongly Liberal
